# Evaluating the social acceptability of the Re‐Connect concept: A smartphone‐based, nonfinancial, contingency management intervention

**DOI:** 10.1002/jaba.2923

**Published:** 2024-11-18

**Authors:** Bethany R. Raiff, Caitlyn R. Upton, Mikhail N. Koffarnus

**Affiliations:** ^1^ Department of Psychology Rowan University Glassboro NJ USA; ^2^ Department of Family and Community Medicine University of Kentucky Lexington KY USA

**Keywords:** acceptability, contingency management, self‐report, smartphones

## Abstract

Contingency management is a well‐validated behavior change intervention; however, the financial incentives can prevent it from being widely adopted. Most Americans have a smartphone with applications (apps) that they find enjoyable and engage with for a considerable amount of time. A potential avenue for contingency management dissemination is a mobile smartphone application that leverages the existing reward value of smartphone apps as a tool for behavior change. The present study examined the acceptability of the Re‐Connect concept, which proposes to block nonessential but highly preferred apps and unlock them contingent on meeting the user's health goals. Out of the sample surveyed (*N* = 146), 63.02% reported that they would be likely to use Re‐Connect and 67.81% would recommend it to someone. Acceptability of Re‐Connect increased with greater user control. These results suggest that access to preferred smartphone apps could be a socially acceptable incentive in a contingency management intervention.

In the United States, 17% of adults report engaging in at least three risky health behaviors, such as smoking, alcohol use, and unprotected sex, with an average of at least one risky health behavior throughout the population (Fine et al., 2004). For teens, 29.8% reported alcohol use, 19.80% reported marijuana use, and 15.40% reported physical activity engagement of under 1 hr in a week (Kann et al., 2019). Improving health behavior can benefit individuals; however, health behavior is notoriously difficult to change and maintain (Kelly & Barker, 2016).

Contingency management (CM) is a behavior‐analytic intervention that involves delivering a reward (usually monetary) contingent on objective evidence of desired behavior change. This strategy has been effective across a range of health behaviors. Prior research has demonstrated the effectiveness of CM in reducing substance use, including cigarette smoking (Coleman et al., 2024; Dallery et al., 2007, 2017; Prendergast et al., 2006), increasing physical activity (Andrade et al., 2014; Irons et al., 2013; Strohacker et al., 2014; Washington et al., 2014), and motivating individuals to lose weight (Jeffery et al., 1978; Thorndike et al., 2016). However, the feasibility of implementing CM interventions remains a challenge when using financial rewards due to barriers associated with funding the incentives (Kirby et al., 2006). Because of this limitation, CM is not readily available to people seeking to change their behavior and is not typically reimbursed by health insurance companies. Nonfinancial alternatives have been researched throughout the years, such as voucher‐based CM programs for smoking cessation that used community donations to provide rewards (Amass & Kamien, 2004), and there is evidence for the effectiveness of CM when using other types of rewards such as material (e.g., bus voucher, bicycle), behavioral (e.g., take‐home doses of methadone), or token rewards (e.g., prize draws) (Corepal et al., 2018; Olmstead & Petry, 2009; Stitzer et al., 1979). For example, among adults enrolled in methadone treatment for opioid use disorder, which requires daily attendance at the clinic to receive the medication, take‐home doses of methadone have been effectively used as incentives to reduce benzodiazepine use (Stitzer et al., 1979). Finding innovative and inexpensive rewards that appeal to a large segment of the population could bring the benefits of CM to more people who need it, without the barriers associated with funding and delivering monetary incentives.

Access to smartphones is a reinforcer for a large portion of the general population. In a 2019 Pew Research Center survey, it was reported that 96% of Americans owned a cellular phone in the United States, 81% of which are smartphones. Americans check their phone once every 12 min, on average, and 88% of the time spent on phones is spent in smartphone applications or “apps” (Buildfire, 2024). People use their phones to access a wide variety of apps, with 96% of people using their phones for messaging apps, 81% for games, 70% for social networking, 47% for retail, and 41% for news (Buildfire, 2024). The high percentage of users who report using apps on their phones suggests that they find engagement with apps reinforcing. Therefore, a CM intervention using access to apps that users already enjoy as rewards is an innovative new direction for research that has the potential to make a large public health impact.

The proposed concept is an app‐blocking‐based CM intervention called “Re‐Connect” that would limit participants' access to high‐valued, but nonessential, phone apps (e.g., games, social media, shopping) until objective evidence of specific health behavior conditions has been met. In Re‐Connect, the user would first agree to block access to one or more of their preferred nonessential apps, rendering them unusable. Next, they would receive tokens when they meet verifiable health goals (e.g., low carbon monoxide for smoking abstinence, steps or active minutes on an activity tracker for physical activity, zero breath alcohol content for alcohol abstinence). These tokens could then be used to unlock their preferred apps for a specific amount of time. For instance, if a participant used Re‐Connect to increase their physical activity, the app could be synced to their Fitbit (or other activity tracker) to verify their steps. This user could have a prespecified step goal to meet and would earn a token when that step goal was reached. That token could then be redeemed for a predetermined period of access to their preferred app(s). The theoretical basis for Re‐Connect relies on the Premack principle—making access to a high‐probability behavior (e.g., preferred smartphone app use) contingent on engaging in a low‐probability behavior (e.g., exercise) to increase the likelihood of engaging in that low‐probability behavior in the future (Klatt & Morris, 2001; Premack, 1959, 1961).

Re‐Connect is based on the well‐supported theoretical and empirical evidence for CM and the Premack principle, but it is important to assess the social validity of such an intervention before developing and testing it (Wolf, 1978). Social validity is often assessed with three domains: (a) significance; (b) appropriateness; and (c) meaningfulness of the goals, procedures, and effects achieved through the intervention (Wolf, 1978). In the current study, we designed and distributed a survey to assess the social validity of the proposed Re‐Connect application. After assessing the overall acceptability of Re‐Connect as we envisioned it, we asked participants to evaluate different features of the application to determine whether changes in user control would influence acceptability (e.g., user‐defined goals, apps blocked, duration of time the apps were unblocked).

## METHOD

### Participants

Participants (*N* = 274) were primarily recruited through the Rowan SONA system (i.e., undergraduate Introduction to Psychology students) and social media sites, such as Facebook. Adults at least 18 years old who had a smartphone were eligible to participate in the study. The mean age at the time of participation was 26.01 years (*SD* = 10.79). Participants who did not complete the acceptability portion of the survey were not included in the analyses, leaving 146 participants. Table 1 includes participant demographics.

**TABLE 1:** Sample demographics.

Demographic variable	*n*	%
Sex/Gender		
Female	72	51.42
Male	56	40.00
Transgender	1	0.71
Neither Female/Male	10	7.14
Prefer not to answer	1	0.71
Race		
White	107	76.43
Mixed race	17	12.14
Black or African American	9	6.43
Asian	6	4.29
American Indian/Alaskan Native	1	0.71
Ethnicity		
Hispanic	17	12.14
Not Hispanic	123	87.86
Education		
No schooling completed	1	0.71
Grade 9–11	1	0.71
High school graduate	15	10.71
Some college	68	48.57
Associate's degree	11	7.86
Bachelor's degree	32	22.86
Master's degree	7	5.00
Doctorate degree	2	1.43
Professional degree	3	2.14
Job		
Employed	56	40.00
Out of work	13	9.29
Retired	3	2.14
Self‐employed	4	2.86
Student	63	45.00
Unable to work	1	0.71

*Note*: Data are from 140 participants who completed the demographics portion of the survey.

### Materials

#### Re‐Connect acceptability

An acceptability questionnaire was developed to ask about specific features and characteristics of the proposed Re‐Connect app. This questionnaire included items to (a) identify preferred target behavior for change, (b) evaluate proposed features of the app, and (c) indicate whether they would recommend this approach to a family member or friend. The questionnaire included a combination of rating scales and multiple‐choice responses. Rating scale responses were given on a 5‐point scale: 5 = *extremely likely*, 4 = *somewhat likely*, 3 = *neither likely nor unlikely*, 2 = *somewhat unlikely*, and 1 = *extremely unlikely*. Multiple‐choice responses included a range of health behaviors for which participants might use or recommend the app including (a) reducing alcohol use, (b) reducing cannabis use, (c) quitting smoking, (d) quitting vaping, (e) increasing exercise/physical activity, (f) losing weight, (g) improving medication adherence/managing medication, (h) managing someone else's behavior (a child, family member, etc.), (i) none, and (j) “other” with a textbox for adding additional targets. In total, there were 18 questions used to assess the acceptability of the proposed program.

#### International Physical Activity Questionnaire (IPAQ)

The eight‐item IPAQ (Hagströmer et al., 2006) assessed engagement in physical activity. The survey separated physical activity into subsets of intensity including vigorous (e.g., heavy weightlifting, fast bicycling, aerobics), moderate (e.g., carrying light loads, bicycling at a normal pace, doubles tennis), walking, and sitting. Participants were asked to report the number of days (0–7 days) they engaged in the activity as well as the amount of time they engaged in the activity in hours and minutes (e.g., During the last 7 days, how many days did you walk for at least 10 minutes?). The IPAQ was scored by first calculating the minutes per week the participant reported engaging in each activity and then by calculating the metabolic equivalent (MET) minutes (amount of energy expended during an activity), where minutes for vigorous activity were multiplied by 8, moderate activity by 4, and walking by 3.3. These scores were then translated into three categories of physical activity: high (1,500 MET min of vigorous activity or 3,000 MET min when combining all activity types), moderate (at least 600 MET min when combining all activity types), and low (below 600 MET min), as defined by Hagströmer et al. (2006).

#### Fagerström Test for Nicotine Dependence (FTND)

The FTND (Heatherton et al., 1991) was administered if the participants indicated that they smoked cigarettes. On the FTND, participants rated their answers to questions assessing nicotine dependence using multiple‐choice questions with a score ranging 0–10. A score of 0–2 indicated very low dependence, 3–7 indicated moderate dependence, and 8–10 indicated very high dependence.

#### Alcohol Use Disorders Identification Test–Consumption (AUDIT‐C)

If a participant reported drinking, the AUDIT‐C (Babor et al., 2001; Bush et al., 1998) was used to assess the likelihood of hazardous drinking. This brief test has been validated for identifying problematic drinking patterns (Bush et al., 1998). The questions are answered using a 4‐point scale, with a total score range of 0–12. A score of 3 or more (females) or 4 or more (males) represents potentially problematic use (Bush et al., 1998).

#### Patient Health Questionnaire (PHQ‐9)

The nine‐item PHQ‐9 (Kroenke et al., 2001) assessed symptoms of depression over the past 2‐week period. These included symptoms such as “little interest or pleasure in doing things”; “feeling down, depressed, and hopeless”; and “poor appetite or overeating” evaluated on a 4‐point Likert scale (*not at all, several days*, *more than half the days*, *nearly every day*).

#### General Anxiety Disorder questionnaire (GAD‐7)

The seven‐item GAD‐7 (Spitzer et al., 2006) assessed symptoms of anxiety over the last 2 weeks, with symptoms including “feeling nervous, anxious, or on edge”; “trouble worrying”; and “being so restless it's hard to sit still” evaluated on a 4‐point Likert scales (*not at all*, *several days*, *more than half the days*, *nearly every day*).

#### Demographics and participant characteristics

We administered a demographic survey consisting of the items described in Table 1. Additional participant characteristics useful for developing the Re‐Connect program were also collected such as what smartphone models people used, their interest in social features, and what behavior participants may like to target for change.

### Procedure

After obtaining informed consent, participants were asked to watch a 2‐min 23‐s video that described the concept of Re‐Connect (See Appendix for the script). This video used animated clip art to explain the concept of Re‐Connect, using physical activity as an example target behavior. A transcript was also provided for participants who may be visually impaired or preferred written information to visuals. Participants were then asked to complete the questionnaires, including the custom acceptability questionnaire. Table 2 includes the acceptability questions.

**TABLE 2:** Percentage of participants endorsing each item on the rating scale and associated statistics.

	1	2	3	4	5	*Mdn* (IQR)	Chi‐square	Odds ratio (CI)
**General Acceptability Questions**								
(1) Based on how we described Re‐Connect, how **likely** would **you** be to use it to meet your health goals?	7.53%	10.96%	18.49%	49.32%	13.70%	4 (3–4)	N/A	N/A
(2) How likely would you be to recommend it to a friend/family member to meet their health goals?	2.05%	6.85%	23.29%	47.95%	19.86%	4 (3–4)		
**Re‐Connect features**								
(3) How likely would you be to use Re‐Connect if **Re‐Connect set the goals** for you?	8.22%	15.07%	20.55%	43.84%	12.33%	4 (3–4)	χ^2^(1, 140) = 17.19*	1.97 (1.4–2.7)
(4) How likely would you be to use Re‐connect if you were able to **set your own** goals?	2.05%	10.96%	18.49%	46.58%	21.92%	4 (3–4)		
(5) How likely would you be to use Re‐Connect if it **randomly blocked the apps** so you don't know ahead of time which ones will be blocked (not including essential apps such as the phone or GPS)?	21.23%	20.55%	20.55%	26.71%	10.96%	3 (2–4)	χ^2^(1, 140) = 35.61*	3.27 (2.2–4.8)
(6) How likely would you be to use Re‐Connect if **you were able to choose the app(s)** that are blocked (not including essential apps such as the phone or GPS)?	3.42%	8.22%	20.55%	49.32%	18.49%	4 (3–4)		
(7) How likely would you be to use Re‐Connect if it unlocked the app for a **specific amount of time** , but not the entire day, after meeting your health goal?	10.96%	22.60%	27.40%	28.08%	10.96%	3 (2–4)	χ^2^(1,140) = 29.05*	2.61 (1.8–3.7)
(8) How likely would you be to use Re‐Connect if it unlocked the app for the **entire day** after meeting your health goal?	6.16%	6.85%	24%	37.67%	25.34%	4 (3–4.25)		

*Note*: The first question in each block describes features as they were presented in the video. *Denotes significant differences in acceptability according to the GEE model, with *p* < .001. Rating Scale: 1 = *extremely unlikely*, 2 = *somewhat unlikely*, 3 = *neither likely nor unlikely*, 4 = *somewhat likely*, 5 = *extremely likely*. Mdn = median; IQR = interquartile range; CI = confidence intervals; GPS = global positioning system.

### Data analysis

The acceptability of the Re‐Connect prototype presented in the video was evaluated using a within‐subject ordinal generalized linear model with generalized estimating equations (GEE) to control for within‐subject effects, an exchangeable correlation matrix, a robust estimator, and a logit link function to accommodate the ordinal Likert‐ and rating‐scale outcome data. Participants' likelihood of using Re‐Connect was compared across three manipulations: (a) Re‐Connect setting goals versus the user setting goals, (b) Re‐Connect randomly selecting applications to block versus the user selecting which applications to block, and (c) earning apps back for a finite amount of time (e.g., 4 hr) versus earning apps back for the entire day. Additional ordinal GEE regression models assessed whether high scores on the PHQ‐9 and GAD‐7 entered as covariates affected the associations between the manipulations and likelihood of use, and an ordinal regression with logit link function assessed wither initial endorsement of the likelihood of using Re‐Connect was associated with PHQ‐9 or GAD‐7 scores. For all ordinal GEE regressions, odds ratios were calculated to assess effect size. In an ordinal regression with binary predictors like in the present experiment, the odds ratio refers to the odds that a participant selected a higher category on the ordinal (Likert‐ or rating‐scale) outcome associated with one of the predictors. It does not specify the amount or degree to which a response was higher because such a value cannot be reliably quantified for ordinal outcomes, just that the response was one or more categories higher (e.g., *somewhat likely* vs. *extremely likely*). Descriptive statistics were calculated for all acceptability items to determine the likelihood that participants would use the proposed program under various conditions. All analyses were conducted in SPSS Statistics 29.

## RESULTS

### Acceptability data

Table 2 includes acceptability questions and data. To assess the acceptability of Re‐Connect, three pairs of features were compared, as noted earlier: (a) Re‐Connect setting goals versus the user setting goals, (b) Re‐Connect randomly selecting applications to block versus the user selecting which applications to block, and (c) earning apps back for a finite amount of time (e.g., 4 hr) versus earning apps back for the entire day. Given the original description of Re‐Connect (see Appendix), 49.32% of participants reported being somewhat and 13.70% reported being extremely likely to use the app to help them meet their health goals; furthermore, 47.95% reported that they would be somewhat and 19.86% reported that they would be extremely likely to recommend it to a friend or family member.

When comparing features, participants were 1.97 (95% CI [1.4–2.7]) times more likely to endorse a higher acceptability rating on the rating scale when choosing their own goals (67.81% *somewhat* or *extremely likely*) over Re‐Connect setting their goals for them (56.17% *somewhat* or *extremely likely*; χ^2^(1, 140) = 17.19, *p* < .001). Participants were 3.27 (95% CI [2.2–4.8]) times more likely to endorse a higher acceptability rating when choosing their own apps to block (67.81% *somewhat* or *extremely likely*) versus having Re‐Connect randomly block their apps (37.67% *somewhat* or *extremely likely*), χ^2^(1, 140) = 35.61, *p* < .001. Finally, participants were 2.61 (95% CI [1.8–3.7]) times more likely to endorse a higher acceptability rating when having the app unblocked for the entire day (63.01% *somewhat* or *extremely likely*) versus having the app unblocked for a specific amount of time (39.04% *somewhat* or *extremely likely*), χ^2^(1, 140) = 29.05, *p* < .001. The PHQ‐9 and GAD‐7 were not found to predict endorsement beyond the app features compared. Thus, in all cases greater user control resulted in higher ratings of acceptability than greater Re‐Connect control.

### Preferred targets for Re‐Connect

Table 3 depicts outcomes for which goals participants indicated they would be interested in targeting with Re‐Connect. The majority of participants, regardless of sex or other demographic variables, endorsed exercise as a target behavior (74%), followed by losing weight (56%). All the other categories (i.e., medication adherence, targeting other people's behaviors, cannabis use, vaping, smoking) were endorsed to a lesser extent.

**TABLE 3:** Percentage of endorsement of target behaviors.

Target	Overall (*N* = 133)		Female (*n* = 81)		Male (*n* = 52)		
	*n*	%	*n*	%	*n*	%
Exercise	99	74.43	65	80.24	34	65.38
Lose weight	74	55.64	51	62.96	23	44.23
Medication adherence	26	19.55	18	22.22	8	15.38
Other people's behavior	31	23.31	16	19.75	15	28.85
Reduce cannabis	21	15.79	11	13.58	10	19.23
Quit vaping	19	14.29	9	11.11	10	19.23
Reduce alcohol	15	11.28	7	8.64	8	15.38
Quit smoking	12	9.02	4	4.93	8	15.38

*Note*: Data are from 133 participants who completed the acceptability portion, the AUDIT‐C, FTND, IPAQ, and demographic section. It should be noted that more people reported “quit smoking” as a goal than reported smoking cigarettes. Total values can exceed 133 because participants could endorse more than one target behavior. AUDIT‐C = Alcohol Use Disorders Identification Test–Consumption; FTND = Fagerström Test for Nicotine Dependence; IPAQ = International Physical Activity Questionnaire.

Further analyses were conducted to determine whether participants who reported using electronic cigarettes (vaping), having a high body‐mass index (BMI), or engaging in risky health behaviors as indicated by the AUDIT‐C (drinking), FTND (cigarette smoking), and IPAQ (low levels of physical activity) identified goals associated with these characteristics for behavior change using Re‐Connect. Table 4 includes the results for these analyses. For example, for all participants who reported vaping, 81.25% of them (*n* = 13 of 16) reported that they would like to use Re‐Connect to target vaping reduction. Only four participants reported cigarette smoking, and all four were classified as having high nicotine dependence on the FTND. Of these four, only one indicated interest in using Re‐Connect to quit. It should be noted that more participants endorsed “quit smoking” as a health goal than endorsed smoking tobacco/cigarettes on the FTND (Table 3). It is possible that these participants also considered other substances (such as marijuana or vaping) to fit broadly under “smoking” when choosing this goal. Finally, although 81 participants reported hazardous levels of drinking, only 14.81% (*n =* 12) selected reducing alcohol use as a target for Re‐Connect. BMI was calculated for all participants who reported height and weight in the demographics section of the survey (*n* = 139). Participants were sorted into categories based on the BMI score: underweight was a score below 18.5, normal weight was a score ranging 18.5–24.9, overweight was a score ranging 25–39.9, and obese was a score of 40 and above. Of the participants who reported an overweight BMI calculation, 74.54% (*n* = 41 of 55) indicated losing weight as a goal. Results also showed that 66.66% of obese weight (*n* = 4 of 6), 41.66% of normal weight (*n* = 30 of 72), and 33.33% of underweight (*n* = 2 of 6) participants indicated losing weight as a goal.

**TABLE 4:** Correspondence between self‐reported health behaviors and endorsed target behavior for Re‐Connect.

Self‐reported behavior	Total	Identified as target	%
Vaping	16	13	81.25
Moderate physical activity	44	35	79.55
Low physical activity	27	21	77.78
High physical activity	62	43	69.35
Smoking	4	1	25.00
Hazardous drinking	81	12	14.81
Overweight BMI	55	41	74.54

*Note*: Data are from 133 participants who completed the acceptability, AUDIT‐C, FTND, IPAQ, and demographic questionnaires. BMI = body mass index; AUDIT‐C = Alcohol Use Disorders Identification Test–Consumption; FTND = Fagerström Test for Nicotine Dependence; IPAQ = International Physical Activity Questionnaire.

## DISCUSSION

Overall, the model of Re‐Connect that was presented in the video featuring health goals set by Re‐Connect, blocked apps chosen by Re‐Connect, and earning back time through meeting health goals were rated as moderately acceptable, with 62.9% of participants indicating that they would be somewhat or extremely likely to use Re‐Connect as it was presented in the video and 65.7% indicating that they would be somewhat or extremely likely to recommend Re‐Connect to a friend or family member. Overall, the data support the concept of Re‐Connect as a novel approach to a CM intervention that arranges access to smartphone applications contingent on meeting objective evidence of health behavior change. Given these outcomes, a prototype of the Re‐Connect application has been developed and testing the efficacy of Re‐Connect is currently underway.

There were significant differences in acceptability among feature variations, with participants favoring features that allowed them more control over Re‐Connect. Participants indicated higher endorsement of Re‐Connect when they were told that they could set their own goals, choose which apps would be blocked, and unlock blocked apps for the full day once health goals were met. The largest difference was found between control over which apps would be blocked, where 67.2% of participants were likely to use Re‐Connect if they had control over what was blocked versus only 37.1% when Re‐Connect had control over what was blocked.

These results are consistent with previous health psychology literature on perceived control (Armitage & Conner, 2001; McEachan et al., 2011). In the health psychology literature, perceived control encompasses an individual's perception of environmental or personal factors that influence whether they can perform a behavior and their perception of the difficulty of the behavior itself (Ajzen, 2002). Participants in the current study favored more control over the factors influencing their behavior (i.e., which apps are being blocked) as well as their perception of the difficulty of their target behaviors (i.e., whether Re‐Connect sets the goals or the user does) are consistent with these two facets of perceived control.

Although participants in the current study favored more control, most CM interventions have historically set goals for participants' smoking (Coleman et al., 2024; Dallery et al., 2007, 2017; Prendergast et al., 2006), physical activity (Andrade et al., 2014; Irons et al., 2013; Strohacker et al., 2014; Washington et al., 2014), and weight loss (Jeffery et al., 1978; Thorndike et al., 2016). This discrepancy opens the opportunity for future directions in combining the principles of perceived control with what is already known about the science of behavior change. Participants in a behavior change intervention are more likely to comply with self‐management interventions when they can select their own goals instead of being assigned goals (Olson et al., 2011), which is consistent with the opinions reported in the present study. Additionally, literature on self‐reinforcement suggests that individuals base their evaluation of their performance on the standards of a model first (Bandura, 1976). For instance, a student may look to a teacher for reference on how to evaluate their work or a dieter may look to a nutrition guide to evaluate their eating habits. An added dialogue about health goals set within a CM framework may be appealing to those seeking to change their behavior or to help people choose the most effective goals. These findings imply that perceived control over elements of Re‐Connect may increase interest and encourage adherence.

It should also be noted that Re‐Connect is different from traditional CM interventions because it falls under the umbrella of aversive control in the form of negative reinforcement (i.e., meeting health goals removes the block on access to their smartphone application) rather than positive reinforcement (i.e., meeting the target behavior results in the delivery of a financial incentive). For this reason, determining the social acceptability of the approach and exploring strategies to increase its acceptability are critical. Unlike with traditional CM, users will always have the option to delete the Re‐Connect app and immediately gain access to the reinforcers being blocked.

Many individuals indicated that they would be interested in using Re‐Connect to pursue a health goal that aligned with a current unhealthy behavior pattern (Table 4), although the degree to which this pattern held varied by behavior. Among individuals who reported vaping, 81.25% identified quitting vaping as a target behavior. Conversely, of participants who were identified by the AUDIT‐C as engaging in hazardous drinking, only 14.81% identified reducing alcohol use as a goal. These results may be reflective of the health goals of the college student sample in this study. High rates of alcohol use are seen in college students (National Institute on Alcohol Abuse and Alcoholism, 2021), and hazardous levels of alcohol consumption are often viewed as normative and an expected part of the college experience (Tan, 2012). Other populations without these patterns may be more likely to identify reductions in hazardous drinking as a target behavior. The most frequently endorsed target behavior was physical activity. It is possible that this is a more universal target for people, especially college‐aged, or it may have been an artifact of the video participants watched to learn about Re‐Connect that used physical activity as an example.

Limitations of this study are worth noting. First, self‐reported measures of social validity were the only targets. Although self‐reported acceptability is a promising first step, more concrete measures of acceptability are needed (e.g., Re‐Connect app downloads, Re‐Connect app engagement). Second, the functional relations between access to smartphone apps contingent on objectively verified target behaviors have yet to be demonstrated. Although access to smartphone apps was not shown as a reinforcer in the current study, there is reason to believe that it can function as a reinforcer. For example, behavioral economic purchasing tasks have been used to demonstrate that demand for smartphone access is high and, in some cases, demand is even higher for smartphones than for food (O'Donnell & Epstein, 2019; Stinson et al., 2021). Third, most of the data were collected for this study during the COVID‐19 pandemic, and the study was therefore conducted online among a convenience sample of college students. The predominately White, college‐aged composition of the sample limits the generalizability of these results. Fourth, although we asked people if they would target a particular behavior with Re‐Connect, we did not ask participants if they were actively seeking treatment for the respective target behavior. In other words, they may have provided low endorsement for any intervention that was not specific to Re‐Connect (related to the above discussion about college student alcohol use). Additionally, 11% of the 146 participants did not complete the entire survey, resulting in a smaller sample for demographic analysis.

In conclusion, the core concept of Re‐Connect was deemed moderately acceptable by participants and therefore merits future investigation. Participants in this study favored more personal control in the health behavior regimens they would use with Re‐Connect, and research in this area should take this into consideration if CM is to be widely distributed in a self‐management format. Future research should also evaluate the efficacy and feasibility of this approach to determine if it can be a viable strategy for promoting behavior change among smartphone users.

## CONFLICT OF INTEREST STATEMENT

Mikhail Koffarnus and Bethany R. Raiff have a conflict of interest because they have the potential to benefit from sales of the final Re‐Connect mobile application.

## ETHICS APPROVAL

This study received institutional review board approval from Rowan University and was conducted in accordance with established ethical guidelines for the treatment of human participants.

## Data Availability

The appendix contains the script and a link to the video that were used to introduce participants to the Re‐Connect concept. Survey responses are available upon reasonable request.

## APPENDIX A

### RE‐CONNECT VIDEO SCRIPT AND LINK

In our lives we have a lot of “I want tos”. I want to start running I want to stop smoking, I want to, I want to, I want to. But moving from “I want to” to “I am” is a totally different ballgame. There are a lot of things stopping you from meeting those goals, such as work, family, friends, and time you probably spend online using social media, playing games, reading, and so on.

So, what if there was an app that helped you meet your health goals by using those things you already love? We are a team of behavioral scientists who are developing Re‐Connect, a smart‐app blocker that can help you meet your “want to” goals by allowing you to use the apps on your phone—such as social media, games, music—only if you meet your health goals.

For example, you could choose running as your goal, pair Re‐Connect with a Fitbit, and then Re‐Connect will set goals for you based on your current daily activity. Re‐ Connect would then keep track of your app usage patterns and learn what apps you might miss the most. Re‐Connect would then pick one or more of those apps at random and block them until you met your goals. As you work harder, Re‐Connect would allow you to earn back time on your apps and provide you with rewards that get better as you meet your goals.

We would love to hear your feedback on Re‐Connect, so please fill out the survey below. Your answers will help us fine‐tune the development of Re‐Connect and make it even better for you. Thank you, and we're looking forward to helping you Re‐Connect with what's important to you.

Link: https://youtu.be/lUNf5SU3JIw
